# Novel plant microRNAs from broccoletti sprouts do not show cross-kingdom regulation of pancreatic cancer

**DOI:** 10.18632/oncotarget.27527

**Published:** 2020-04-07

**Authors:** Xi Xiao, Carsten Sticht, Libo Yin, Li Liu, Svetlana Karakhanova, Yefeng Yin, Christina Georgikou, Jury Gladkich, Wolfgang Gross, Norbert Gretz, Ingrid Herr

**Affiliations:** ^1^Molecular OncoSurgery Group, Section of Surgical Research, Department of General, Visceral and Transplant Surgery, University of Heidelberg, Heidelberg, Germany; ^2^Medical Research Centre, Medical Faculty Mannheim, University of Heidelberg, Mannheim, Germany; ^*^These authors contributed equally to this work and share the first authorship; ^#^These authors contributed equally to this work and share the last authorship

**Keywords:** broccoli, broccoletti, Brassica rapa sylvestris, plant microRNAs, cross-kingdom regulation

## Abstract

Food-derived plant microRNAs are suggested to control human genes by “cross-kingdom” regulation. We examined microRNAs in sprouts from *Brassica rapa sylvestris*, known as broccoletti, which are widely used as sulforaphane supplements, and assessed their influence on pancreatic cancer. RNA was isolated from 4-day-old sprouts, followed by deep sequencing and bioinformatic analysis. We identified 2 new and 745 known plant microRNA sequences in the miRbase database and predicted 15,494 human target genes and 76,747 putative 3′-UTR binding sites in these target genes. The most promising candidates were the already known microRNA sequence bra-miR156g-5p and the new sequence Myseq-330, both with predicted human target genes related to apoptosis. The overexpression of the respective oligonucleotides by lipofection did not alter the viability, apoptosis, clonogenicity, migration or associated protein expression patterns in pancreatic cancer cells. These data demonstrate that broccoletti sprouts contain microRNA sequences with putative binding sites in human genes, but the sequences evaluated here did not affect cancer growth. Our database of broccoletti-derived microRNA sequences provides a valuable tool for future analysis.

## INTRODUCTION

Pancreatic ductal adenocarcinoma (PDA) is one of the most lethal malignancies, causing the fourth leading cancer-related mortality of both men and women in the Western world [1]. The lack of effective treatment options, especially in the advanced stage [2], is due to a pronounced chemo- and radio-resistance and a usually late diagnosis. Recent data suggest that the broccoli-derived bioactive agent sulforaphane may enhance the therapeutic efficacy of cytotoxic therapy. Sulforaphane weakens cancer stem cells, which are considered the bases of tumour growth and metastasis [3–6]. Currently, there is no drug with purified sulforaphane. Therefore, many companies offer freeze-dried broccoli (*Brassica oleracea*) or broccoletti (*Brassica rapa sylvestris*) sprouts, which usually contain 20–100-fold more sulforaphane than unprocessed broccoli [7].

**Figure 1:**
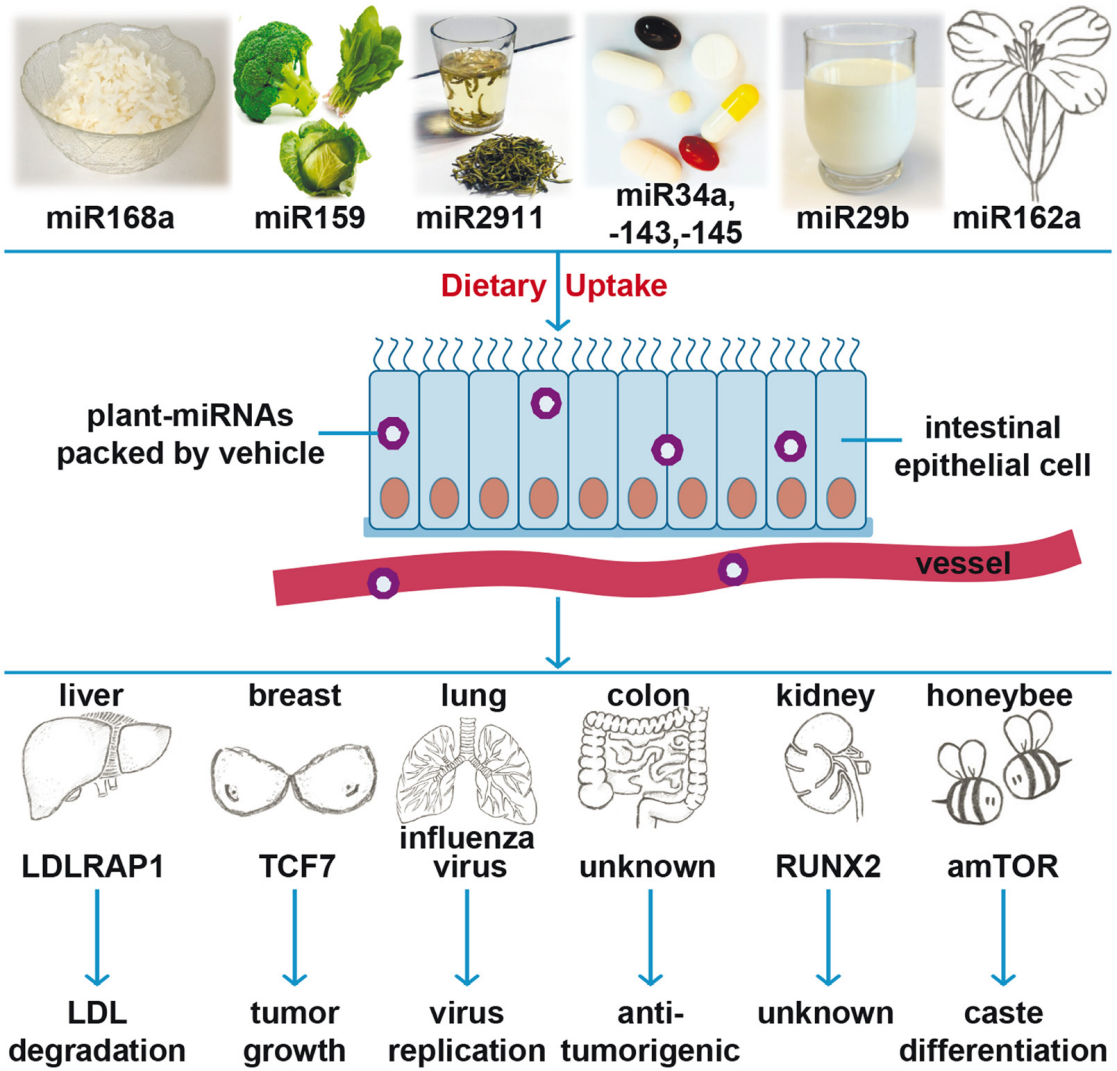
Cartoon of recent findings of plant miRNA actions. Recent studies suggest that the dietary uptake of miR-168a from rice, miR-159 from broccoli, miR-2911 from honeysuckle, a cocktail of plant-miRs (-34a, -143 and -145), miR-29b from cow milk, and miR-162a from *Brassica campestris* safely pass the gastrointestinal tract and can be found in the blood stream or tissue of consumers. Cross-kingdom regulatory effects of plant-derived miRs in other species have described: miR-168 effects low-density lipoprotein removal by targeting low-density lipoprotein receptor adapter protein (LDLRAP1); miR-159 inhibits breast cancer growth by targeting transcription factor 7 (TCF7); miR2911 suppresses viral infection by inhibiting virus replication; a cocktail of plant-miRs (-34a, -143 and -145) reduces colon tumour burden through an unrevealed mechanism; miR-29 targets runt-related transcription factor 2 (RUNX2); and miR-162a regulates honeybee caste development by targeting mTOR.

However, broccoli and broccoletti may provide more than just sulforaphane to fight cancer. Several recent studies in a wide range of organisms have demonstrated that exogenous microRNAs (miRs), mainly derived from food, stably pass the gastrointestinal track and enter the bloodstream [8, 9]. Exogenous miRs are suggested to regulate the expression of target genes in other species, and this phenomenon is called cross-kingdom regulation [10–15]. For instance, miR168a from rice bound to human or mouse low-density lipoprotein receptor adapter protein 1 (LDLRAP1) mRNA and reduced the removal of low-density lipoprotein from plasma (Figure 1) [10]. Similarly, broccoli-derived miR159 was found in human serum, and the abundance of miR159 was inversely correlated with breast cancer incidence and progression in patients [11]. Both miR168a and miR159 were resistant to sodium periodate oxidation and cooking [11]. The high stability of most plant miRs was attributed to the methylation of their 3′ ends [16]. Similarly, miR2911, isolated from honeysuckle, was detectable in the serum and lungs of mice fed with honeysuckle [12]. The presence of miR2911 in serum inhibited the replication of influenza type A viruses *in vitro* and *in vivo* [12]. Moreover, the oral application of a cocktail of mouse-derived tumour suppressor miR sequences (miR34a, miR143, miR145), modified with a methyl group typical for plant-miRs, was able to block the tumourigenesis of colon cancer in mice [13]. Furthermore, the administration of cow´s milk to 5 healthy volunteers or mice led to an increase in miR29b and its target gene RUNX2 in plasma. Although the sequences of bovine and human miR29b are identical, miR29b derived from milk exosomes is able to enter the human intestine before it is released into the blood stream [14]. Additionally, the presence of the plant miR162 in the pollen and honey diet of honeybees seems to play a role in the development of worker bees or queen bees [15]. These findings led to the hypothesis that the cross-kingdom regulation of human gene expression by dietary miRs from other species may be a conserved biological process. However, this hypothesis is controversial and widely questioned because of faulty experimental controls [17], contaminated probes or mismapping [18, 19], and the fact that the uptake of miRs from diet has never actually been shown directly. Some labs have subsequently found extensive contamination of the human samples, such as the Wilmes lab, who contributed to the initial studies [20], and other labs have retracted their papers because positive results were based on misinterpretations and technical errors [21, 22]. Only two labs [10, 12, 14] have been able to produce consistent positive results, but in each case, the findings have been controversial because other studies have failed to reproduce the data [17, 23].

Despite some similarities, plant and animal miRs exert large differences in many aspects of their location, biogenesis, and function. For example, animal miRs are mostly encoded within introns, while most plant miRs are located in non-protein-coding transcription units [24]. Although both miR types are formed by cleavage from pri-miRs and pre-miRs, the location of biogenesis and the responsible enzymes are different, which explains why animal miRs are usually 22 to 23 nucleotides long, whereas plant miRs are shorter and usually contain only 21 nucleotides [24]. As mentioned above, the 3′ ends of plant miRs are usually methylated, which confers stability, but only a few animal miRs have the same modification. Usually, plant miRs are absolutely complementary to their target mRNAs, while animal miRs adopt partial complementarity for target recognition. Based on differences in mRNA and miR interactions, plant miRs induce target mRNA degradation, while animal miRs mainly repress translation [25].

The present study examined whether broccoletti sprouts, which are commonly used as sulforaphane supplements, contain plant miR sequences, and if these sequences impact the progression of PDA. A total of 747 mature sequences of broccoletti-miRs were identified. This included 2 novel and 745 previously known plant miR sequences. The candidate sequences were examined by *in silico* analysis, which led to the identification of the top two candidates with target genes involved in apoptosis signalling. The present study sheds new light on the ongoing debate of miR-induced cross-kingdom regulation and provides a valuable platform for future analysis of the function of broccoletti sprout-derived miRs.

## RESULTS

### Identification of 747 plant miR mature sequences in broccoletti sprouts

**Figure 2:**
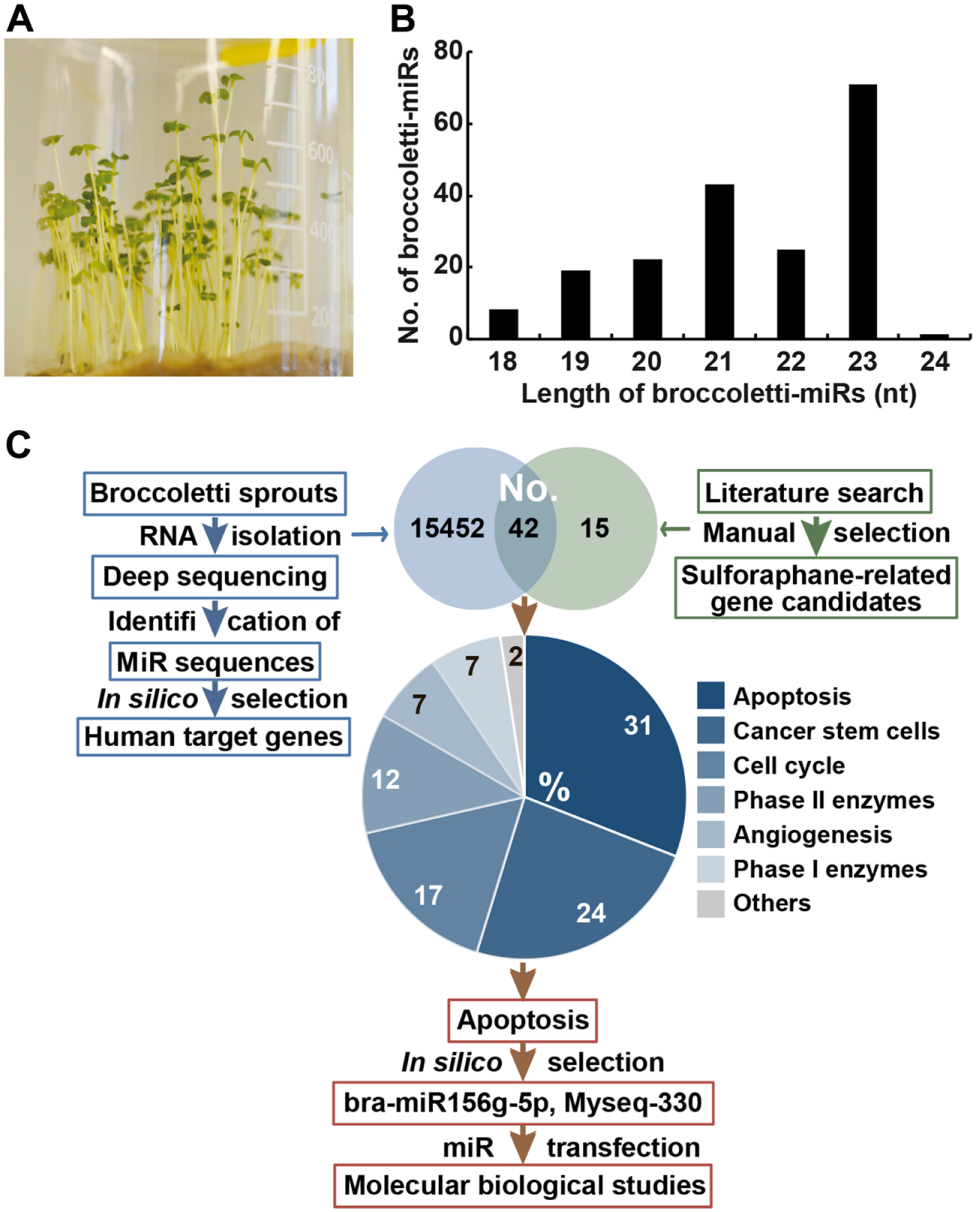
Identification of 747 miR sequences in broccoletti sprouts. (**A**) Broccoletti seeds were incubated with sterile water in a beaker with sterile cotton at the bottom. A photograph of the 4-day-old sprouts is shown. (**B**) RNA was harvested from 4-day-old sprouts, and the sequences of broccoletti-miRs were identified by deep sequencing. The lengths of mature broccoletti-miR sequences from 18 nucleotides to the maximal length of 24 are shown in relation to the number (No.) of respective broccoletti-miRs. Lengths of 21, 22, or 23 nucleotides were most common. (**C**) Strategy for the selection of top broccoletti-miR candidates. We performed two search strategies: (I) the identification of sulforaphane-related (green) human target genes by a literature search and (II) the identification of broccoletti-miR-related (blue) human target genes by *in silico* analysis of putative broccoletti-miR binding sites in human target genes. In this way, we identified 42 human genes common to both search strategies, and their number (No.) is shown in the Venn diagram. The target genes of the highest percentage group with 31% had a function in the apoptosis pathway. Two broccoletti-miR top candidates, the known bra-miR156g-5p and Myseq-330 (similar to aly-miR166b-3p), were identified, which are suggested to have apoptosis regulatory function.

Broccoletti seeds from *Brassica rapa sylvestris,* also known as *Brassica rapa cymosa,* Italian broccoli, broccoli di rape, cime di rape, or rapini, with a sulforaphane content of 8.1 g/kg were cultivated (Figure 2A). RNA was extracted from 4-day-old sprouts and used for deep sequencing. The data were analysed with the online database miRDP1.3 (miRDeep-p), which resulted in the identification of 747 miR mature sequences (Supplementary Table 1). We found that 40% of broccoletti-miRs were approximately 23 nucleotides long, and the overall length varied from 18 to 24 nucleotides (Figure 2B). Only 34 broccoletti-miRs contained the typical uracil at the 5′-end. Next, we matched the obtained broccoletti-miR sequences with known plant-miR sequences of the *Brassica rapa* species, to which broccoletti belongs. According to the online database miRBase [26], 96 *Brassica rapa* miRs have been registered and verified so far. Seventy-eight of the registered miRs matched our detected broccoletti-miR sequences, while 18 miRs were mismatched (Supplementary Table 1). Because some plant-miRs are highly evolutionarily conserved between different species [27], we downloaded 10,898 plant-miR datasets of 128 plants from the plant-miR database [28] and compared these sequences with our data. Surprisingly, 5,872 datasets matched 745 of our broccoletti-miR sequences, while only two of our sequences were novel, broccoletti-specific sequences. These novel sequences are referred to as Myseq-248 and Myseq-578 according to the Myseq system [29], where “Myseq” symbolizes “My sequence” and “X” is a unique identifier. For the detected broccoletti sequences that map previously registered *Brassica rapa* miRs, we used the mirBase nomenclature, and for the additional detected broccoletti sequences, we used “Myseq-X”. More specifically, several sequences of the miR159 family (e. g., bna-miR159, far-miR159) matched the broccoletti-miRs Myseq-258, Myseq-439, and Myseq-643, and their sequences were identical to the known miR159 sequence 5′-UUUGGAUUGAAGGGAGCUCUA-3′, which was detected in mature broccoli [11]. The miR159 family is involved in leaf shape, flowering and floral transition among *arabidopsis* [30], rice [31] and *Sinningia speciosa* [32]. Similarly, our Myseq-10 sequence matched bol-miR824, aly-miR824 and ath-miR824. The Myseq-10 sequence belongs to a conserved biogenesis plant pathway, and ath-miR824 was shown to contribute to flowering repression by targeting the gene AGAMOUS-LIKE16 in *Arabidopsis* [33].

### Prediction of human targets of the identified broccoletti-miR candidates

To obtain knowledge on the regulatory function of the identified broccoletti-miRs in pancreatic cancer, we applied Target Prediction for miRs (TarPmiR) software [34]. In this way, 15,494 human target genes were found to harbour putative binding sites for 747 broccoletti-miRs within their 3′-UTRs (Supplementary Table 2). Among these broccoletti-miRs, the broccoletti-miR Myseq-420 had the highest number of human target genes of 8,662, while the broccoletti-miR Myseq-602 had only 67 targets. By GSEA, we identified human genes belonging to a specific pathway and detected 206 broccoletti-miRs, which play a role in 280 human pathways (Supplementary Table 3). Among the identified genes, the most significant genes encoded proteoglycans, which play a pivotal role in pancreatic tumourigenesis, progression, metastasis, immune response and chemo-resistance [35]. Moreover, 10 cancer pathways were enriched in our prediction, which are common for cancer of the bladder, breast, colorectum, endometrium, stomach, prostate, pancreas, thyroid and lung. Additionally, most of the other pathways were directly or indirectly correlated with pancreatic cancer. For instance, Myseq-412 (bra-miR860-3p) was predicted to regulate the ascorbate and aldarate metabolism, which induces cell death and autophagy in pancreatic cancer [36]. Similarly, broccoletti-miR Myseq-424 (gra-miR8785) is correlated with focal adhesion and bra-miR156g-5p is correlated with adherens junctions and migration upon dysregulation. Equally, Myseq-621 (zma-miR169a-3p) is associated with renin secretion, and its inhibition improved the clinical outcome of advanced pancreatic cancer [37]. Additionally, Myseq-229 (bra-miR172a) was associated with glutamine metabolism, which is important for pancreatic cancer growth [38]. Additionally, Myseq-474 (aly-miR164a-3p) was predicted to influence FoxO signalling, which is targeted by sulforaphane [39].

### Selection of three strong broccoletti-miR candidates involved in apoptosis

To further limit the number of broccoletti-miR candidates to the most important miRs, we related their candidate genes to sulforaphane targets. By using a PubMed search, we identified 1,730 manuscripts related to sulforaphane. Among these papers were 241 reviews and 698 cancer-related and 207 inflammation-related manuscripts. By manual selection, we listed 57 genes involved in apoptosis, cell cycle, detoxification and self-renewal (Supplementary Table 4). These genes were compared with the 15,494 human target genes of the identified broccoletti-miRs. A Venn diagram shows that 42 genes are targets of both broccoletti-miRs and sulforaphane (Figure 2C). Among these genes, 31% were involved in apoptosis, 24% in cancer stem cell signalling, 17% in cell cycle regulation, 12% in detoxification by phase II enzymes, 7% in angiogenesis, 7% in detoxification by phase I enzymes and 2% in other functions. For experimental evaluation, we selected genes from the largest group, with 13 genes related to apoptosis, and the most corresponding broccoletti-miRs were bra-miR156g-5p and Myseq-330 (similar to aly-miR166b-3p) (Table 1).

**Table 1:** Broccoletti-miR candidates and selected human target genes

Candidates	Apoptosis-related targets	Sulforaphane-related targets
bra-miR156g-5p	AKT1, AR, CASP3, CASP8, FOXO1, FOXO4, TP53, XIAP	CDK6, CDKN1A, CTNNB1, CYP1B1, IL6R, KDR, MAPK1, SMO, ZEB1
Myseq-330 (aly-miR166b-3p)	AR, CASP8, FOXO1, FOXO3, FOXO4, HDAC6, TP53, XIAP	CCND1, CTNNB1, GJA1, HIF1A, IL6R, KDR, MAPK1, MYC, POU5F1, REL, STAT3

Among a huge amount of human target genes, we selected the apoptosis- and sulforaphane-related target genes of the broccoletti-miR candidates bra-miR156g-5p and Myseq-330.

### Broccoletti-miRs can be detected after lipofection in PDA cells

**Figure 3:**
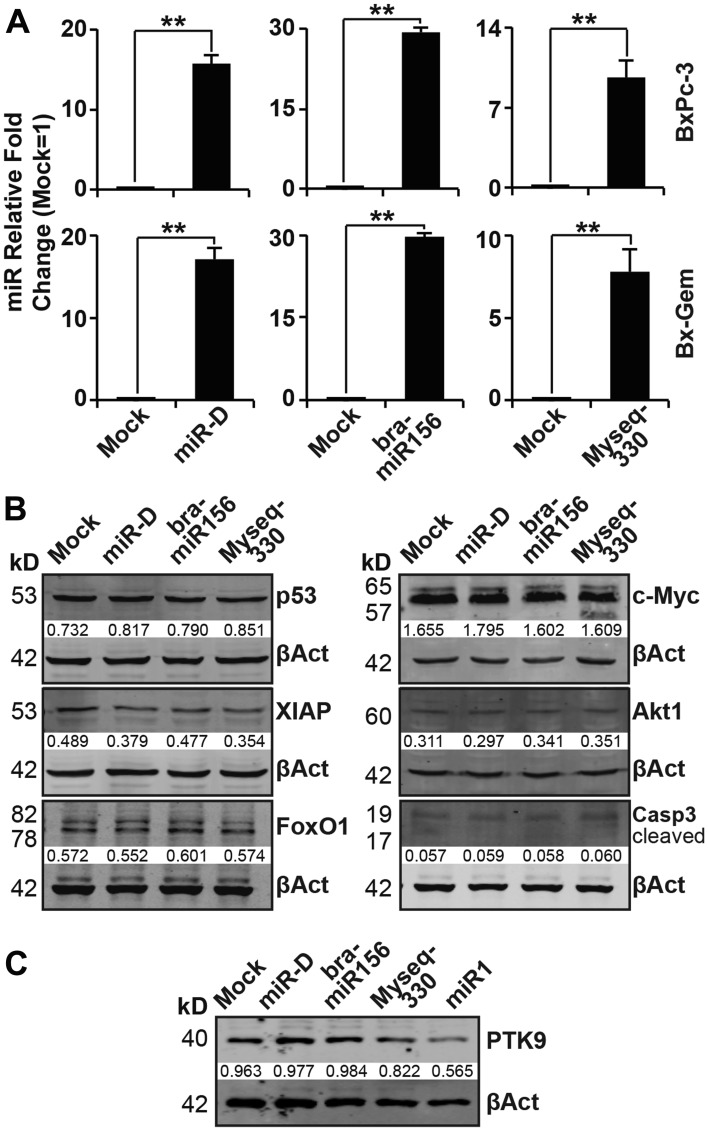
Lipofection of broccoletti-miRs in PDA cells does not induce the expression of target genes. (**A**) BxPc-3 and Bx-Gem cells were transfected with bra-miR156g-5p, Myseq-330, miR-D (50 nM each), or a miR-NG oligonucleotide (50 nM), which served as a mock control. At 24 h after transfection, the RNA was isolated, followed by reverse transcription. The samples were validated by TaqMan^®^ miR assay. RNU44 was used to normalize the expression level, and the average fold change of the mock control was set to 1. The relative fold changes ×1000 are presented. Experiments were performed in triplicate, and the data are shown as the means ± SD (^**^ *P* < 0.01). (**B**) Proteins were harvested from BxPc-3 cells at 24 h after lipofection, and western blot analysis was performed for p53, XIAP, FoxO1, c-Myc, Akt1 and caspase-3. β-actin served as a control for equal loading conditions. The protein sizes are shown in kilodaltons (kD). The band intensities were measured using ImageJ and are shown below the bands. The band intensities were normalized to β-actin. (**C**) Total proteins were harvested from BxPc-3 cells at 24 h after lipofection, and PTK9 protein levels were evaluated via western blot analysis and examined as described above.

Mimics of bra-miR156g-5p and Myseq-330, as well as a designed-miR, were lipofected into the PDA cell lines BxPc-3 and Bx-Gem. Here, we arbitrarily selected a designed-miR (similar to stu-miR8005c) as a plant-derived miR control, with the sequence 5′-UCCAAGGGUUUAGGGUUUAGGGA-3′, which was predicted to regulate apoptosis. A nonsense miR mimic was used as a control. After 24 h, RNA was harvested, and TaqMan probe-based qRT-PCR was performed. The results demonstrated an extremely high expression of all specific miR mimics in both cell lines compared to that in the control, which was set to 1 (Figure 3A). To examine the effect of our 2 broccoletti-miR candidates and the designed-miR on protein expression, we performed western blot analysis and detected the protein expression of the candidate genes caspase-3, XIAP, FoxO1, p53, c-Myc and Akt in transfected BxPc-3 cells (Figure 3B). However, no effects were found, since all bands looked equal. Therefore, we verified our conditions and lipofected the positive control miR1. As expected, the expression of its target gene protein tyrosine kinase 9 (PTK9) was significantly downregulated at 24 h after the lipofection of 50 nM of the miR1 mimic (Figure 3C). This result suggests that our lipofection method worked.

### Broccoletti-miR candidates do not influence basal and induced apoptosis

**Figure 4:**
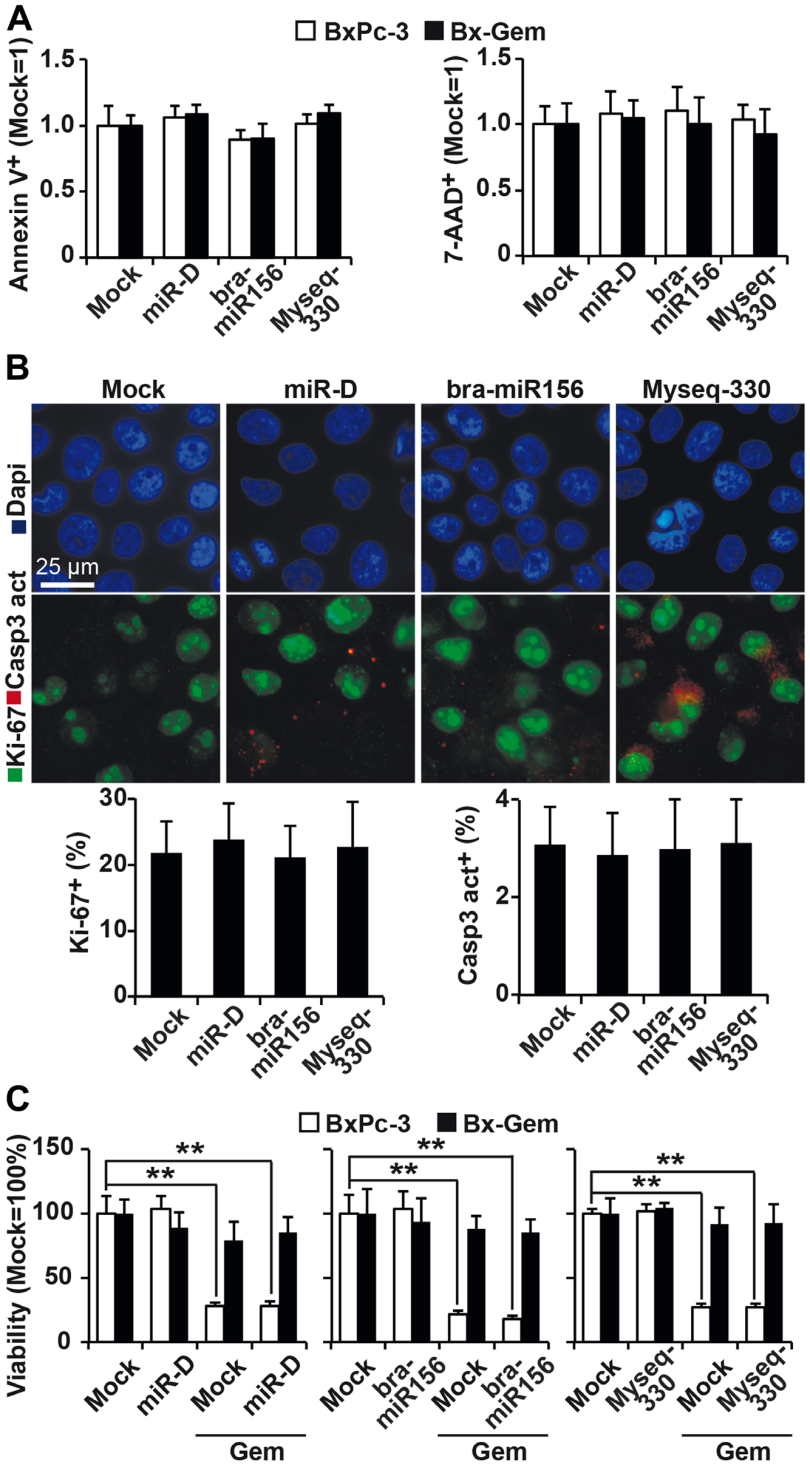
Lipofection of top broccoletti-miR candidates does not influence basal and induced apoptosis. (**A**) BxPc-3 and Bx-Gem cells were transfected as described in Figure 3. Seventy-two hours later, the cells were stained with Annexin V-PE and 7-AAD, followed by FACS analysis. The percentage of Annexin V- and 7-AAD-positive cells is shown. (**B**) Lipofected BxPc-3 and Bx-Gem cells were stained with an antibody specific for the proliferation marker Ki-67 (green) or the apoptosis marker cleaved fragment of caspase-3 (red), which indicates apoptosis. Representative images at ×100 magnification are shown. The percentage of Ki-67- or caspase-3-positive cells was counted in 18 visual fields, and the means ± SD are shown in the diagram below. (**C**) BxPc-3 and Bx-Gem were lipofected as described above, and at 24 h later, the cells were treated with gemcitabine (10 nM) or were left untreated. Ninety-six hours after gemcitabine treatment, viability was determined by MTT assay. The data are presented as the means ± SD (^**^ *P* < 0.01).

To evaluate other effects of the two broccoletti-miRs and the designed-miR on apoptosis, BxPc-3 and Bx-Gem cells were stained at 1, 2 and 3 days after lipofection with Annexin V and 7-AAD, followed by FACS analysis. No significant differences between the control and treatment groups were found (Figure 4A, Supplementary Figure 1A). Moreover, immunofluorescence staining detected no difference in caspase-3 or Ki-67 expression (Figure 4B, Supplementary Figure 1B). Next, we evaluated whether the broccoletti-miRs or designed-miR might influence chemotherapy-induced apoptosis and treated the transfected cells with gemcitabine or left them untreated, followed by measurement of the viability by MTT assay. Whereas gemcitabine at a concentration of 10 nM significantly reduced the viability at 24, 48, 72 and 96 h after treatment, no effect of broccoletti-miRs or controls was observed (Figure 4C, Supplementary Figure 1C).

### Broccoletti-miR candidates do not affect cell viability and morphology

**Figure 5:**
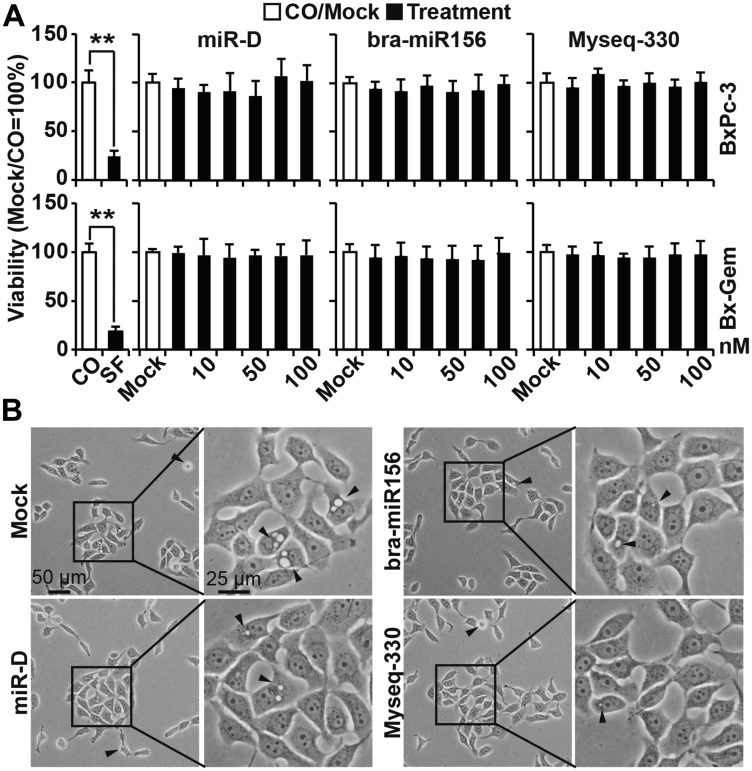
Lipofection of the top broccoletti-miR candidates does not alter cell viability or morphology. (**A**) BxPc-3 and Bx-Gem cells either treated with sulforaphane (SF, 10 μM) or were left untreated (CO). Additionally, the cells were lipofected with increasing concentrations of bra-miR156g-5p, Myseq-330, miR-D from 1 to 100 nM or with the miR-NC Mock control (50 nM). At seventy-two hours later, the viability was measured by MTT assay, and the data are presented as the means ± SD. (**B**) The morphology of BxPc-3 cells was photographed at 24 h after lipofection by phase-contrast microscopy (50 nM broccoletti-miRs, miR-D or miR-NC Mock control). Representative images at ×100 and ×200 magnification are shown. The arrows indicate apoptotic blebbing.

To rule out the possibility that we used a suboptimal concentration of the broccoletti-miRs and therefore did not see an effect, we lipofected 1, 10, 25, 50, 75 and 100 nM of the specific broccoletti-miRs and 25 nM of a control into BxPc-3 and Bx-Gem cells. However, under these conditions, we were not able to detect any significant differences by MTT analysis (Figure 5A, Supplementary Figure 2). This finding is in contrast with that of a control experiment, in which non-transfected cells were left untreated or were treated with sulforaphane for 24, 48 or 72 h. Sulforaphane significantly reduced the viability in both cell lines, as expected. Accordingly, we were unable to detect any obvious morphological differences by microscopy (Figure 5B, Supplementary Figure 3).

### Broccoletti-miR candidates do not affect clonogenicity and migration

**Figure 6:**
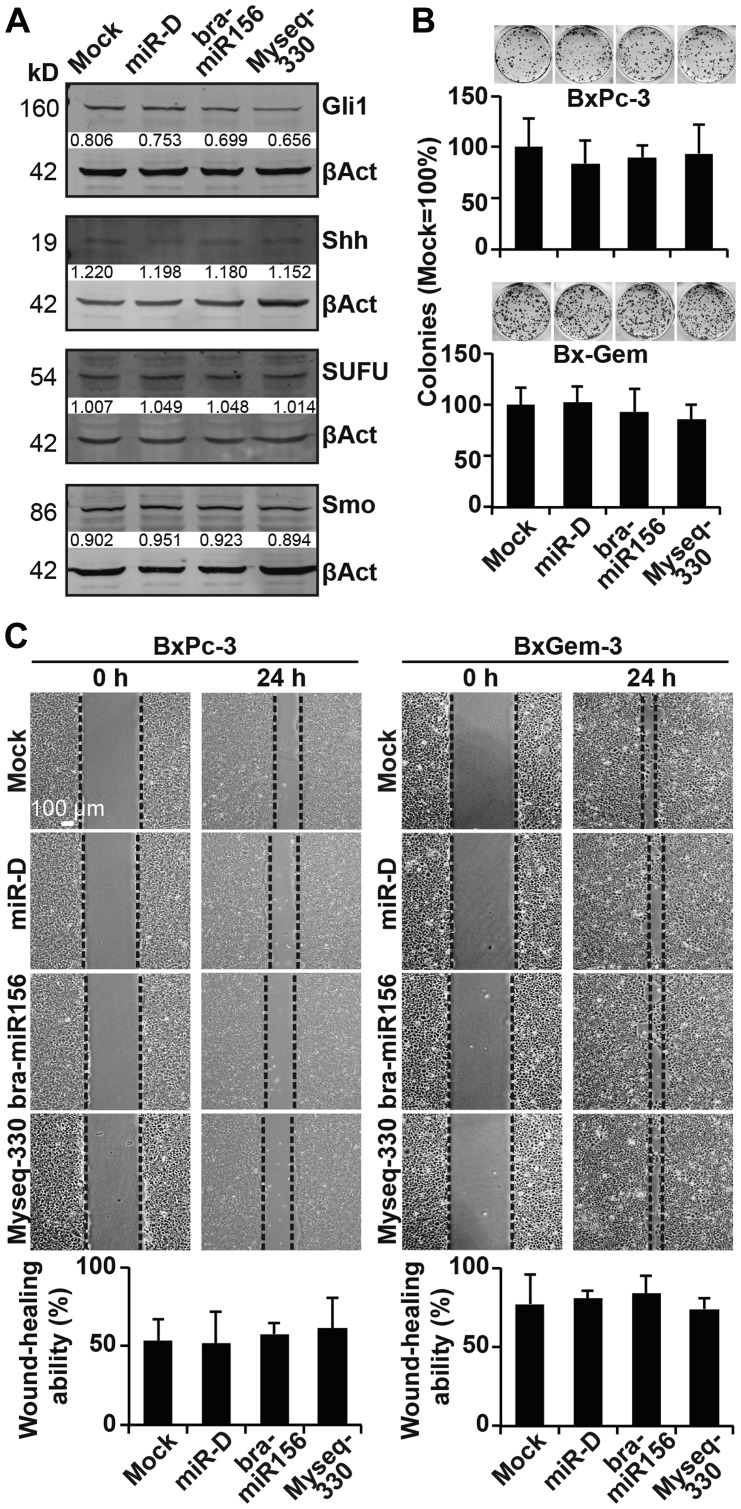
Lipofection of the top broccoletti-miR candidates does not affect clonogenicity and migration. (**A**) Proteins were harvested from BxPc-3 cells as described in Figure 3B, and the expression of Gli1, Shh, SUFU and smoothened was detected by western blot analysis. The protein sizes are shown in kilodaltons (kD), and the band intensities were measured using ImageJ and are shown under the bands. The band intensities were normalized to β-actin. (**B**) Twenty-four hours after lipofection, BxPc-3 and Bx-Gem cells were reseeded at a low density (400 cells/well) and cultured for 2 weeks in regular culture medium. Then, the cells were fixed and stained with Coomassie, followed by the evaluation of colonies comprising at least 50 cells. The number of colonies in the mock group was set to 1. (**C**) Similarly, at 24 h after lipofection, the cells were seeded onto 6-well plates and cultured until they reached 90% confluence. Then, a line was scratched into the middle of the cell layer with a 10-μl pipette tip. The closure of the wounded region was evaluated and recorded by microscopy at ×100 magnification. The size of the gap area was calculated by ImageJ software.

Next, we examined whether our broccoletti-miR candidates may affect cancer progression. Twenty-four hours after the lipofection of BxPc-3 cells, the proteins were harvested, and the expression of the Hedgehog proteins Gli1, Shh, SUFU and smoothened was examined by western blot analysis. However, again, no obvious differences in expression patterns among the treatment and control groups were detected (Figure 6A). Similarly, colony-forming assays or scratch assays with BxPc-3, Bx-Gem, AsPC-1 and PANC-1 did not detect any significant differences (Figure 6B, 6C, Supplementary Figure 4A, 4B).

## DISCUSSION

The detection of plant-miRs in a wide range of organisms, including humans [8, 20], mice [40, 41] and insects [42], as well as the ability of these molecules for cross-kingdom regulation, prompted us to examine the effects of broccoletti sprout-derived miRs on pancreatic cancer progression. We identified 2 novel sequences and 747 mature *Brassica rapa subspecies sylvestris*, also known as Broccoletti, miR sequences, which matched known plant-miR sequences. By prediction analysis, we identified putative human targets of our broccoletti-miRs and downscaled these results to the 2 most promising apoptosis-related candidates. However, we did not find any significant functional effect of these candidates.

To our knowledge, broccoletti sprout RNA has never been sequenced before, in contrast to RNA from other *Brassica rapa* species, as described in publications in the field of botany [43–46]. In these manuscripts, the authors used *Brassica rapa subspecies chinensis* to investigate the miR expression in pollen development [43] and heat stress response [44]. One of the reports used 4-week-old *Brassica rapa subspecies pekinensi* to detect stomatal development [45], and the other report examined the miRs of 14-day-old *Brassica rapa subspecies oleifera* in disease-resistance [46]. We were able to correlate some of our 747 broccoletti-miR sequences with the sequences of *Brassica rapa* miRs (bra-miRs) because these sequences are available in the miRBase online tool. However, despite being members of the same plant species, only 78 bra-miRs matched our detected broccoletti-miR sequences, and 18 bra-miRs were mismatched (Supplementary Table 1). The high number of non-matching broccoletti-miRs may be due to genetic variations between different subspecies *sylvestris* and the other cultivars (e. g., *chinensis, pekinensi, etc.)*, which was used in the miRBase.

Using the TarPmiR program, we forecasted 15,494 cross-kingdom matches of human target genes and determined the most promising broccoletti-miR candidates. However, functional evaluation did not provide any significant differences in pancreatic cancer. One explanation may be the incompatibility between plant and mammalian miR functions. For example, cell temperature and pH differ between plant and mammalian cells, or mammalian cells lack some enzymes and proteins, which are required for the proper binding and splicing of plant-miRs. In this regard, the Dicer and RNA-induced silencing complexes (RISC) differ between these species. For example, the main miR effector in plants to form RISC is AGO1 (Argonaute protein), while mammalian miRs can use AGO1, AGO2 and AGO3 without bias [47].

Moreover, the cross-kingdom hypothesis is controversial. Despite a large number of promising predictions, many studies failed to detect plant-miRs in consumers of a plant-based diet [17, 48, 49], and the authors of these studies have expressed doubts about the cross-kingdom hypothesis. Even if miRs could be delivered through the diet, it might be physically difficult to reach the levels used in cell culture experiments. Even *in vitro*, at supraphysiological levels, and even with highly efficient delivery into cells using lipofection, miRs that are predicted to affect target transcripts often have no measurable effect, as in our case. Additionally, the obvious grave differences between plant and mammalian miRs, concerning genomic structure, biogenesis, matching principle and acting mechanism, may form an extensive biological barrier. The previously indicated interactions between plant miRs and the regulation of genes in other species might randomly be due to specific sequences. Accordingly, the cross-kingdom hypothesis could be a rather rare phenomenon. Moreover, the inability of other researchers to replicate the experiments of the first cross-kingdom regulation study [10] raises additional concerns. With regard to plant miR159, which was shown to target transcription factor 7 (TCF7) in breast cancer [11], we were not able to confirm this prediction. Although our deep sequencing analysis detected the known miR159 sequence (Myseq-258, Myseq-439, Myseq-643), we did not further address the experimental function because our *in silico* analysis did not predict TCF7 as a human target of the plant miR159.

Another topic of discussion concerns our strategy for the prediction of gene targets because we selected only sulforaphane-related genes. The TarPmiR database is a classification model that hardly covers any information for plant sequences; therefore, it is not clear how reliable this prediction can be. Compared to over 2,000 human miRs, which can regulate more than 60% of human genes, the prediction of 15K gene targets of 747 broccoletti-miRs is likely to involve many false predictions, especially because known plant-miRs have distinct sequences with their human homologs.

In conclusion, we found no evidence that broccoletti miR sequences affect predicted human target genes. Our data combined with those of other authors suggest that it is unlikely that dietary RNAs are functional in mammals. However, we were the first to identify sequences of putative miRs from broccoletti sprouts. Even though we were not able to find a biological function for the 2 selected broccoletti-miR candidates in human pancreatic cancer cell lines, our study provides a new database of broccoletti-miRs, which is now available for further studies.

## MATERIALS AND METHODS

### Culture of broccoletti sprouts and RNA extraction

Seeds of the sulforaphane-rich broccoletti prototype *Brassica rapa sylvestris* (Megerle Online GmbH, Ubstadt-Weiher, Germany) were cultivated with distilled water at room temperature. After 4 days of germination, the whole sprouts with roots were gently collected, rapidly frozen in liquid nitrogen and minced with sterile scissors. Total RNA was extracted with the mirVana™ miRNA Isolation Kit and the Ambion^®^ Plant RNA Isolation Aid (both from Life Technologies GmbH, Darmstadt, Germany).

### Deep sequencing

The next generation sequencing of broccoletti-sprout-derived miRs was performed by the CellNetworks Deep Sequencing Core Facility (Bioquant Center, University of Heidelberg, Heidelberg, Germany). One sample was sequenced, and small RNA libraries were constructed using the NEBNext^®^ Small RNA Library Prep Set for Illumina^®^ (New England BioLabs, Ipswich, USA). The total RNA samples were sequenced by a polymerase-based sequence-by-synthesis method on an Illumina HiSeq2000 platform (Illumina, California, USA). The sequencing data are available in the public database for microarray experiments, Array Express (https://www.ebi.ac.uk/arrayexpress/experiments/E-MTAB-7155/).

### *In silico* analyses

The raw deep sequencing results were analysed by miRDeep-p version 1.3 (http://www.mybiosoftware.com/mirdeep-p-1-3-analyzing-the-microrna-transcriptome-in-plants.html) to identify the miR transcriptome of broccoletti sprouts. A plant-specific scoring system was used to filter the data [50]. Bad quality sequences and Illumina adapter sequences were removed with the online quality control tool for high throughput sequence data, FastQC (http://www.bioinformatics.babraham.ac.uk/projects/fastqc/). The broccoletti genome and its annotation files (*Brassica rapa* Annotation Release 101) were downloaded from the National Center for Biotechnology Information (NCBI) and used for mapping with the reads of the broccoletti libraries to identify broccoletti sprout-derived miRs (ftp.ncbi.nlm.nih.gov/genomes/). For comparing our broccoletti-miR sequences with other plant-miRs, the plant-miR databases miRBase 21 [26] and PMRD [28] were used. For the prediction of human target genes, the computer program computer program Target Prediction for miRs (TarPmiR) [34] and the miRanda 3.3a algorithm (http://www.microrna.org/microrna/home.do) were used, and the human genome was downloaded from the NCBI database (ftp. ncbi. nih. gov/genomes/H_sapiens/RNA/rna.gbk.gz). A gene set enrichment analysis (GSEA; https://www.gsea-msigdb.org/gsea/index.jsp) was performed with the computer language “R” (https://rcommand.com/main.html) and the Fast Gene Set Enrichment Analysis Algorithm (fgsea; https://bioconductor.org/packages/release/bioc/html/fgsea.html) to determine the association of certain pathways involving the predicted human target genes (https://bioconductor.org/packages/release/bioc/html/fgsea.html). Pathways belonging to various cell functions were obtained by using the public external database Kyoto Encyclopedia of Genes and Genomes (KEGG, https://www.genome.jp/kegg).

### Tumour cell lines

The human established PDA cell lines BxPc-3, AsPC-1 and PANC-1 were purchased from the American Type Culture Collection (ATCC, Manassas, VA, USA). The gemcitabine-resistant cell line Bx-Gem was established in our laboratory based on BxPc-3 as described previously [6]. All cell lines were authenticated by a commercial institution (Multiplexion, Heidelberg, Germany) and tested monthly for mycoplasma by PlasmoTest™ (InvivoGen, San Diego, USA). All cells were cultured under standard conditions in DMEM supplemented with 10% FCS and 25 mmol/L HEPES (Thermo Fisher, Dreieich, Germany). Gemcitabine was obtained from the Clinic Pharmacy of the University Clinic of Heidelberg, Germany.

### miR transfection

The mirVana™ custom mimics miR-D (designed-miR sequences), bra-miR156g-5p, Myseq-330 (Supplementary Table 1), and miR-positive (miR1) and miR-negative (miR-NC) controls were obtained from Thermo Fisher Scientific (Dreieich, Germany) and transfected with Lipofectamine 2000 (Thermo Fisher Scientific) as described in the manufacturer’s instructions.

### miRNA isolation and qRT-PCR

The miRNeasy^®^ Mini Kit was used according to the manufacturer’s instructions (Qiagen, Hilden, Germany), and the RNA concentrations were measured with a NanoDrop 2000 spectrophotometer (Nano Drop Technologies, Wilmington, USA). cDNA was prepared from 10 ng of total RNA using the TaqMan^®^ miRNA Reverse Transcription Kit (Thermo Fisher Scientific, Dreieich, Germany) according to the manufacturer’s instructions. Quantitative levels of broccoletti-miRs were measured using a relative Custom TaqMan™ assay (Assay ID: miR-D, CTXGP26; bra-miR156g-5p, CTYMJM3; Myseq-330, CT47VTH) and RNU44 as an endogenous control (Assay ID: 001094) from Life Technologies. TaqMan^®^ Universal Master Mix (without UNG) (Thermo Fisher Scientific) and the StepOne Real-Time PCR System (Applied Biosystems, Darmstadt, Germany) were used for the PCR. Primer sequences are available upon request from Thermo Fisher Scientific.

### Viability assay

The cell viability was measured by 3-(4,5-dimethylthiazol-2-yl)-2,5-diphenyltetrazolium bromide (MTT) as described previously [51].

### Colony forming assay

The cells were lipofected with miR mimics for 24 h and then re-seeded onto 6-well plates (400 cells/well) in triplicate, followed by incubation for 14 days without changing the cell culture medium. After fixing with 3.7% paraformaldehyde, staining with 0.05% Coomassie blue, washing and overnight drying, the number of colonies comprising at least 50 cells was counted under a microscope as described previously [4].

### Wound healing analysis

The cells were lipofected with miR mimics, followed by trypsinizing and re-seeding at 5 × 10^5^ cells per 6-well plate until 70~80% confluence was reached. Then, a cross was scraped into the cell layer with a 10-μL pipette tip, followed by gently washing twice with 1× PBS and then adding fresh culture medium supplemented with 1% FCS. After 24 h of incubation, images of the wound healing area were obtained by microscopy, and the open area was evaluated by ImageJ software.

### Western blot analysis

The proteins were harvested, and western blot analyses were performed as described previously [4]. The following antibodies were used: Rabbit monoclonal antibodies (Abs) from Cell Signaling Technology, Inc. against p53 (cat. no. 2527; dilution, 1:1,000), X-linked inhibitor of apoptosis protein (XIAP; cat. no. 2045; dilution, 1:1,000), c-Myc (cat. no. 5605; dilution, 1:1,000), the cleaved fragment of caspase-3 (cat. no. 9664; dilution, 1:1,000), GLI family zinc finger 1 (Gli1; cat. no. 3538; dilution, 1:1,000), SUFU negative regulator of hedgehog signaling (SUFU; cat. no. 2520; dilution, 1:1,000) and sonic hedgehog signaling molecule (Shh; cat. no. 2207; dilution, 1:1,000); rabbit polyclonal Abs from Abcam against smoothened (cat. no. ab72130; dilution, 1:1,000); rabbit polyclonal Abs from Thermo Fisher Scientific, Inc. against protein tyrosine kinase 9 (PTK9; cat. no. PA5-29312; dilution, 1:1,000); mouse monoclonal Abs from Cell Signaling Technology, Inc. against FoxO1 (cat. no. 14952; dilution, 1:1,000) and Akt1 (cat. no. 2967; dilution, 1:1,000); and mouse monoclonal Abs from Sigma-Aldrich (Merck KGaA) against β-actin (cat. no. A1978; dilution, 1:5,000). Membranes were incubated with primary antibodies at 4°C overnight. Positive bands were detected using IRDye Secondary antibodies and the Odyssey^®^ CLx Imaging system (both from LI-COR Biosciences).

### Measurement of apoptosis

The cells were stained with PE-conjugated Annexin V and 7-AAD (Thermo Fisher Scientific, Dreieich, Germany) and analysed by flow cytometry using a FACSCanto™ workstation with BD FACS Canto™ Software Norton Ghost™ (FACSCanto, BD Biosciences, New Jersey, USA).

### Immunofluorescence staining

Immunofluorescence staining was performed with cells grown on sterile glass coverslips (Carl Zeiss Microscope, Oberkochen Germany) in 6-well plates. The cells were fixed with 4% PFA at room temperature for 10 min, and blocked with 10% goat serum (Thermo Fisher Scientific, Dreieich, Germany) in PBS at room temperature for 30 min. Mouse monoclonal Abs against caspase-3 (cat. no. NB100-56708; dilution, 1:200; Novus Bio-Techne GmbH, Wiesbaden Nordenstadt, Germany) and rabbit monoclonal Abs against Ki67 (cat. no. ab92742; dilution, 1:400; Abcam) were used. Secondary antibodies were incubated at RT for 30 min. The secondary Abs for Ki67 were Alexa 488 (dilution, 1:400) and for caspase-3 Alexa 594 (dilution, 1:400; both from A11032; Thermo Fisher Scientific, Inc.). The stained cells were evaluated at a Leica DM RB fluorescent microscope using the SPOT Advanced Version 4.6 software (http://www.spotimaging.com/software/spot-advanced/). The cells were incubated with primary antibodies for 1 h at room temperature.

### Statistical analyses

The quantitative data are presented as the means ±SD of at least three independent experiments. Differences between groups were tested with Student’s *t*-test and corrected for multiple comparisons with the Bonferroni-Holm method by the use of Excel (Microsoft Corporation) and the JMP 14 software (SAS Institute, Inc.). *P* < 0.05 was considered statistically significant. In the gene set enrichment analysis, a false discovery rate (FDR) of 5% was used to adjust for multiple testing.

## SUPPLEMENTARY MATERIALS









## Abbreviations

miRs: microRNAs
PDA: pancreatic ductal adenocarcinoma

## Notes

**Editorial note**

This paper has been accepted based in part on peer-review conducted by another journal and the authors' response and revisions as well as expedited peer-review in Oncotarget.
