# Supplementary material for: Novel plant microRNAs from broccoletti sprouts do not show cross-kingdom regulation of pancreatic cancer
Source: Oncotarget. 2020 Apr 7;11(14):1203–17. doi: 10.18632/oncotarget.27527 (PMC7147085; doi:10.18632/oncotarget.27527)
Supplement: Supplementary file 1 [file oncotarget-11-1203-s001.pdf]

Novel plant microRNAs from broccoletti sprouts do not show cross-kingdom regulation of pancreatic cancer

SUPPLEMENTARY MATERIALS

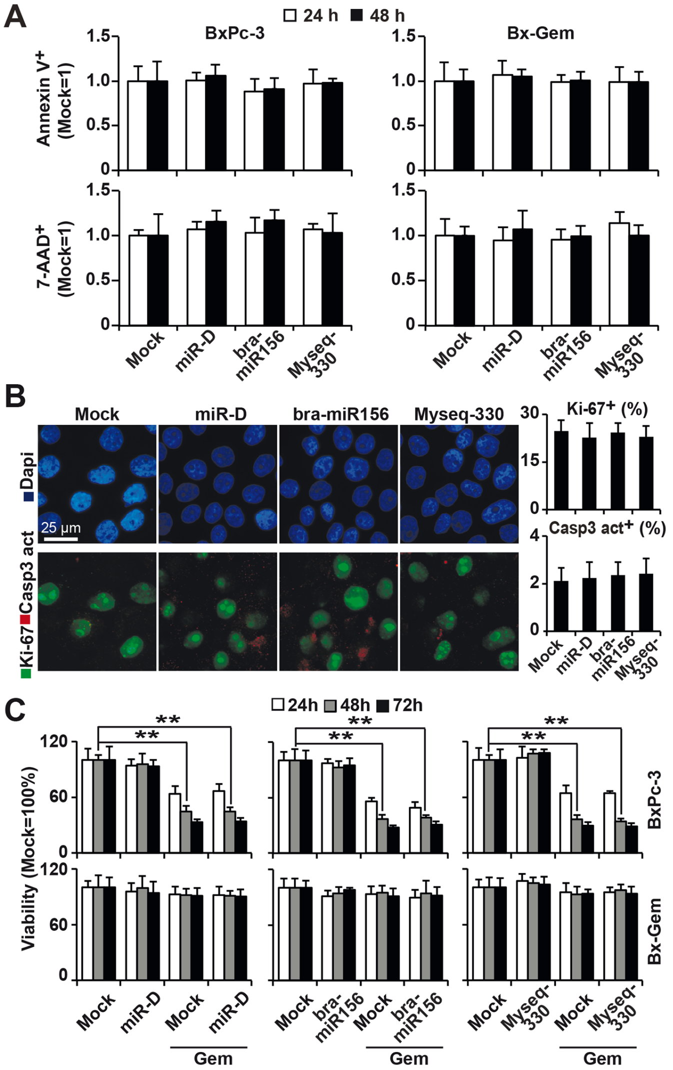

Supplementary Figure 1: Lipofection of top broccoletti-miR candidates does not influence basal and induced apoptosis. BxPc-3 and Bx-Gem cells were lipofected with bra-miR156g-5p, Myseq-330, miR-D (50 nM each), or a miR-NG oligonucleotide (50 nM), which served as a mock control. (**A**) Twenty-four and forty-eight hours later, the cells were stained with Annexin V-PE and 7-AAD, followed by FACS analysis. The percentage of Annexin V- and 7-AAD-positive cells is shown. (**B**) Forty-eight hours after transfection, the BxPc-3 and Bx-Gem cells were stained with an antibody specific for the proliferation marker “Ki-67” (green) or the apoptosis marker “cleaved fragment of caspase-3” (red), which indicates apoptosis. Representative images at ×100 magnification are shown. The percentage of Ki-67- or caspase-3-positive cells was counted in 18 visual fields, and the means ± SD are shown in the diagram below. (**C**) BxPc-3 and Bx-Gem were lipofected as described above, and 24 h later, the cells were treated with gemcitabine (10 nM) or were left untreated. Twenty-four, forty-eight or seventy-two hours after gemcitabine treatment, the viability was determined by MTT assay. The data are presented as the means ± SD ($^{**}P < 0.01$).

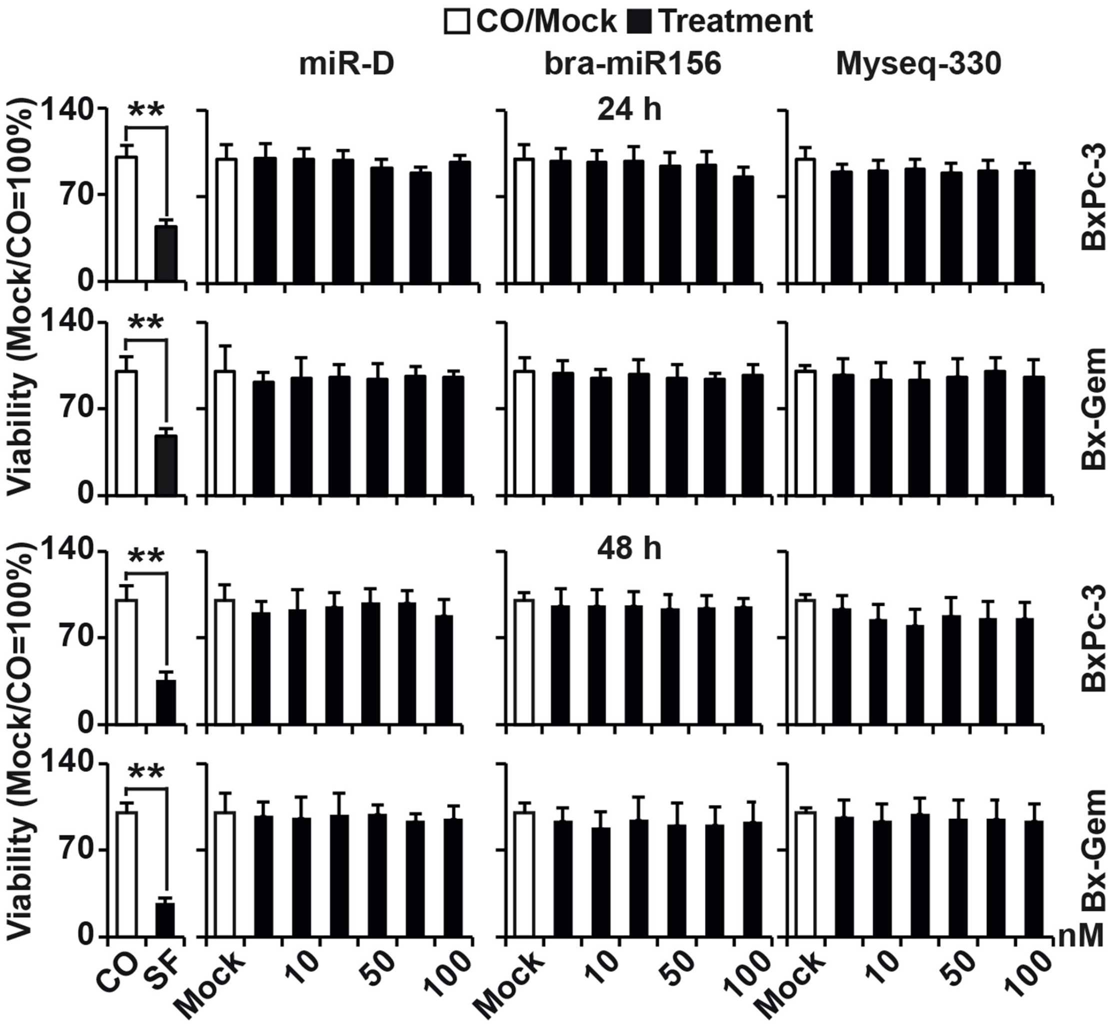

Supplementary Figure 2: Lipofection of the top broccoletti-miR candidates does not affect viability. The cells were treated and analysed as described in Figure 4 and time points are indicated.

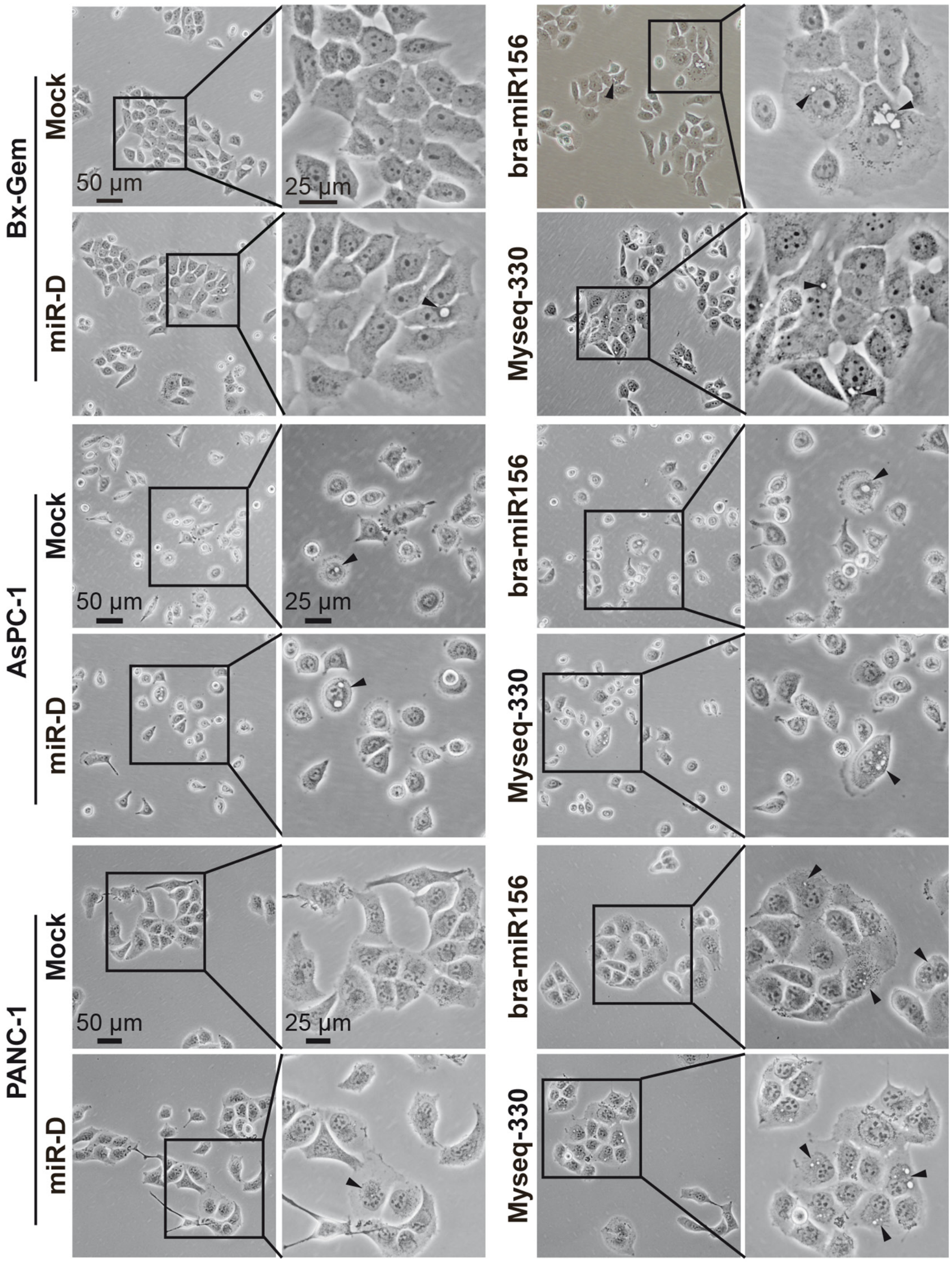

Supplementary Figure 3: Lipofection of the top broccoletti-miR candidates does not affect morphology. The morphology of Bx-Gem, AsPC-1 and PANC-1 cells was photographed at 24 h after lipofection as described in Figure 4. The arrows indicate apoptotic blebbing.

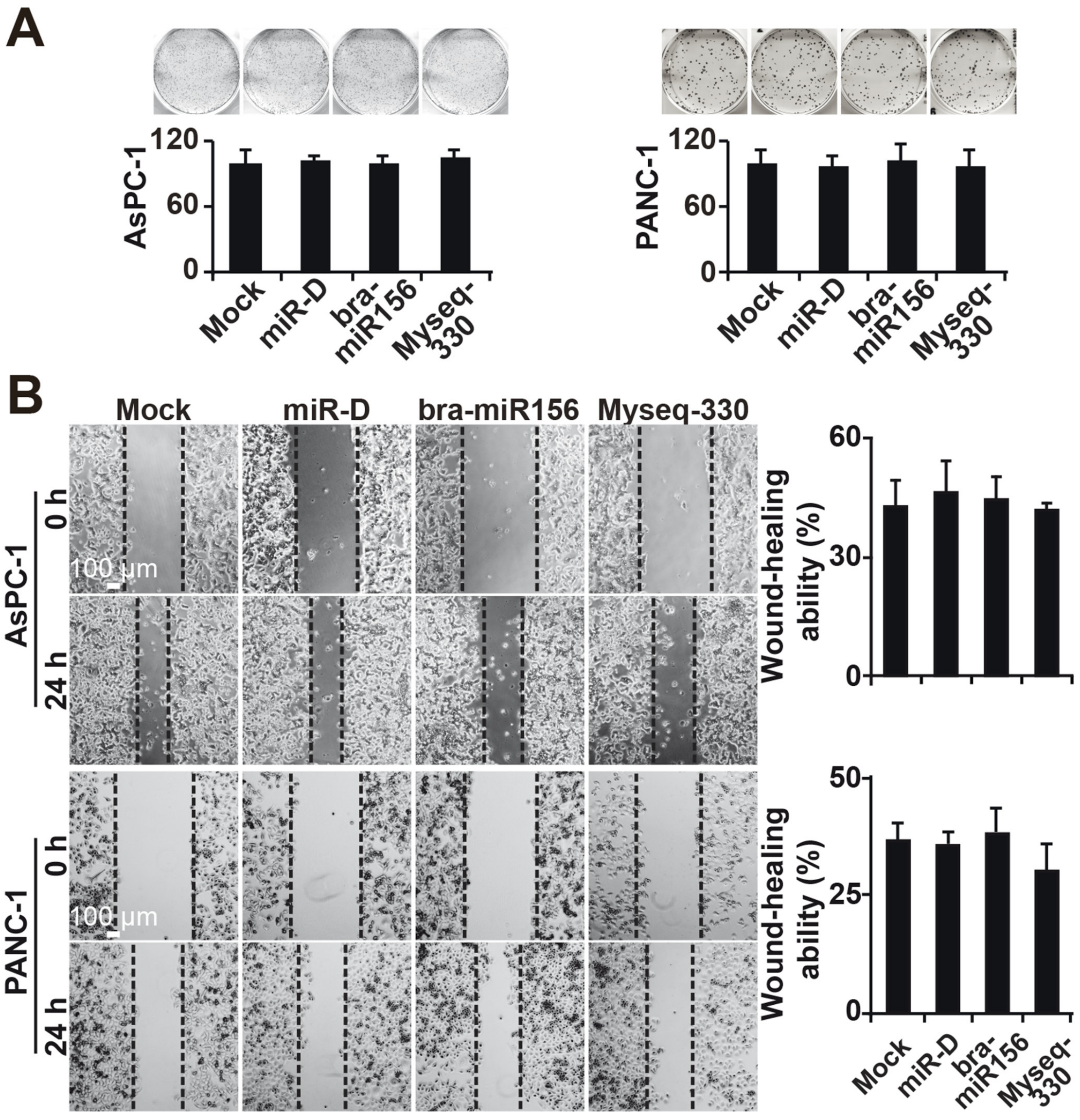

Supplementary Figure 4: Lipofection of the top broccoletti-miR candidates does not affect clonogenicity and migration. The AsPC-1 and PANC-1 cells were treated and analysed as described in Figure 5. The (**A**) colony-forming assays and (**B**) scratch assays were examined, as indicated.

Supplementary Table 1: List of broccoletti sprout-derived miRs (broccoletti-miRs). See Supplementary Table 1

Supplementary Table 2: List of human targets of broccoletti-miRs. See Supplementary Table 2

Supplementary Table 3: Significant pathways suggested to be regulated by broccoletti-miRs. See Supplementary Table 3

Supplementary Table 4: Sulforaphane-related genes

Pathway	Gene name
Angiogenesis	HIF1A, KDR, MYC, VEGF
Apoptosis	AKT1, AR, ATG5, BAX, BCL2, CASP3, CASP8, CASP9, FOXO1, FOXO3, FOXO4, HDAC6, PARP, TP53, XIAP
Cell cycle	CCND1, CDK4, CDK6, CDKN1A, CDKN1B, CHK2, MAPK1, MAPK3, PI3K
Phase I enzymes	CYP1A1, CYP1B1, CYP2E1, CYP3A4
Phase II enzymes	GSTA1, GSTM1, GSTT1, NRF2, UGT1A1, UGT1A8, UGT1A10
Self-renewal and CSC characters	ALDH1, CTNNB1, Gli1, Gli2, IL6R, MMP7, NANOG, NOTCH1, OCT4, PDGFRα, POU5F1, REL, SMO, STAT3, TWIST1, ZEB1
Others	GJA1, HSP90

Genes were identified by literature search and grouped according to their biological function.
